# Rapid, expression‐free bacteriophage‐based specific detection of target bacteria by conditional release of encapsidated reporter molecules

**DOI:** 10.1002/btm2.70171

**Published:** 2026-08-20

**Authors:** Ákos Avramucz, Joseph P. Wheatley, Sahan B. W. Liyanagedera, Tamás Fehér, Richard Amaee

**Affiliations:** ^1^ Synthetic and Systems Biology Unit, Institute of Biochemistry HUN‐REN Biological Research Centre Szeged Hungary; ^2^ Doctoral School in Biology, Faculty of Science and Informatics University of Szeged Szeged Hungary; ^3^ School of Engineering University of Warwick Coventry UK; ^4^ Biophoundry Inc. Chapel Hill North Carolina USA; ^5^ Lucidix Biolabs Chesham, Bucks UK

**Keywords:** bacteriophage‐based diagnostics, capsid‐contained reporter protein, *Escherichia coli* K1, luminescence assay

## Abstract

Rapid diagnosis of infectious diseases is of paramount importance to prevent or control outbreaks and pandemics. Detection of bacteria is commonly performed using culture‐based and molecular detection methods, which cannot address the need for quick, specific and cheap diagnostics. Bacteriophage‐based assays rely on the rapidity, specificity, contaminant‐tolerance and effectiveness of phage‐host interactions and can be engineered with fluorescence or luminescence‐based reporters. Previous attempts, however, required transcription and translation of reporter genes, leading to long assays and restrictive protocols. Here, we shortened the signal generation time by detecting the injection of a phage protein, thereby circumventing the need for gene expression altogether. In our model diagnostic assay, we demonstrate that injection of the N‐terminal fragment of the split nanoluciferase protein of *Oplophorus gracilirostris,* fused to the products of genes g6.7 or g14 of phage K1F, is detectable upon injection into an *Escherichia coli* cell as early as 3 min after phage addition. The engineered phages generate a signal upon exposure to cognate K1—but not to non‐cognate K5 capsule‐enclosed *E. coli* cells, confirming the specificity of our system. The early luminescent signal and the ability to detect as few as 10^4^ bacteria may open the way to the development of a rapid diagnostic tool based on phage‐mediated protein injection.

## Graphical abstract

Schematic of the model diagnostic system. Bacteriophage infection results in the injection of NnLuc protein fragment (N) into the bacterial host. The CnLuc fragment (C) and the substrate are provided exogenously. Lysing the cell allows binding of NnLuc to CnLuc and the substrate, leading to a luminescent signal.

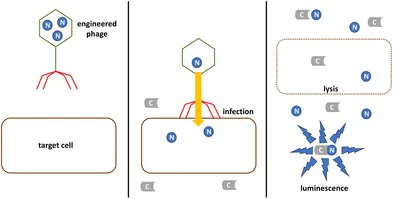

**Translational Impact Statement**

This is the first work that relies on the injection of a proteinaceous agent from a bacteriophage into the host bacterium as the fundamental phenomenon on which detection of the bacterial cell is based. We demonstrate the selectivity of this system, and that it does not require transcription of the injected phage DNA by the host machinery. This technique generates a positive signal in 3 min after phage infection, making it much faster than gene expression‐based bacterial detection techniques.

## 1 INTRODUCTION

One of the first lessons learned during the COVID‐19 pandemic was the important role of rapid and scalable tests in the early diagnosis and isolation of infected individuals. In parallel, the ever‐growing incidence of infections caused by multi‐resistant bacterial pathogens^1^ underlines the need for rapid tests to identify bacteria also, thereby permitting the early initiation of targeted therapeutic interventions. To detect bacteria, both culture‐based and molecular biology‐based methods have been widely used. The former is usually time consuming, and requires further downstream analysis carried out by trained personnel. The latter most often rely on polymerase chain reaction (PCR) or antibody‐based detection. PCR, especially qPCR, also requires highly skilled staff, expensive infrastructure and in practice also ISO quality certification of the testing lab and its staff. While antibody‐based detections are widely applied in bedside test kits, their sensitivity and specificity are often limited. Therefore, there is still a need for a quick, specific and cheap method to detect bacteria in biological samples.

Bacteriophage‐based assays to detect bacteria rely on the rapidity, specificity and effectiveness of phage‐host interactions. Furthermore, phage can tolerate a high degree of sample impurity (with some phage having first been identified in sewage^2^, ^3^, ^4^), which speaks to their potential robustness when deployed in diagnostic kits designed for use by the general public, who lack the training rigor of diagnostic lab staff and may lack willingness for performing manual handling steps. Classical phage amplification assays have long been used to identify various bacterial pathogens, and are based on the ability of one or more bacteriophage (phage) strains to replicate (e.g., form plaques) on the pure culture of the target bacterium. Other phage‐based methods capture target bacteria via tail‐spikes^5^, ^6^, ^7^ or immobilized phage endolysin components.^8^, ^9^, ^10^ These latter techniques however usually require further downstream analysis.^11^, ^12^ The advent of genetically engineered phages made possible the use of assays detecting fluorescent or luminescent signals that indicate phage infection.^13^ These signals are generated by the target cells, after infection by phage, upon expression of the respective phage‐borne reporter transgenes injected by the phages. In the current work, we tested whether the time needed for phage‐based signal detection could be shortened by bypassing expression by the direct detection of a ready‐made luminescent protein, which undergoes ejection from the phage capsid, in response to the presence of its cognate host. This approach can circumvent the need for phage‐borne gene expression as a prerequisite for signal generation—currently the bottleneck in signal detection.

Since we are not aware of signal sequences directing proteins into the capsid of T7‐related phages, we chose to fuse our luminescent reporter protein to intracapsid proteins (ICPs) of the chosen phage. The ICPs (and presumably, their fusion derivatives) spontaneously localize to the inside of the capsid^14^ which prevents them from engaging in premature reactions until their host‐triggered ejection. ICPs have been thoroughly studied in phage T7 and we reasoned that their orthologues in the closely related phage K1F would share enough similarity as to allow T7‐gained knowledge to be applicable.

The first step of phage infection involves the phage successfully binding to its cognate host. In the next step, the ICP proteins are propelled from the phage particle into the host cytoplasm in a defined sequence: gp6.7 and gp7.3 first exit together.^14^ Then, gp14, gp15 and gp16 partially unfold (due to their size exceeding the capsid's aperture) so as to exit the phage nozzle and then spontaneously refold within the host envelope to form the ejectosome: a tubular multi‐protein structure. In this process, gp14 forms the outer channel within the host outer membrane, while gp15 and gp16 form a complex to extend the ejectosome tunnel to traverse the periplasmic space and span the inner membrane. Finally, the phage genome is delivered through the ejectosome into the host cytoplasm, where phage propagation commences.^15^

A potential luminescent reporter, the nanoluciferase protein from *Oplophorus gracilirostris* can be split into an N and a C terminal fragment (NnLuc and CnLuc, respectively), each of which alone lacks activity.^16^ Crucially for this application, these enzyme fragments can *spontaneously* recombine in solution, regaining the ability to emit light in the presence of the substrate compound (here Furimazine). We hypothesized that if the smaller N terminal fragment of nanoluciferase (NnLuc) was fused to an ICP (gp6.7 or gp14), and the C terminal nanoluciferase fragment (CnLuc) and Furimazine were provided exogenously (either in the cell's cytoplasm or outside the cell following lysis), the injection of the NnLuc fusion protein into the host cell during infection could be detected due to the resultant luminescence signal. In essence, the NnLuc fragment, necessary to elicit light in the presence of CnLuc and substrate, would be kept sequestered inside the capsid until and unless a cognate host was supplied.

We display here a proof of principle luminescence detection system where two different modified K1F phages are used to detect the presence of an *Escherichia coli* strain protected by a K1 capsule, and expressing the C terminal nanoluciferase fragment (CnLuc). We also showcase a model diagnostic test system for the detection of wild type bacterial cells by administering the purified CnLuc protein to the cells externally followed by the permeabilization of the cell envelope. We demonstrate that injection of NnLuc into a bacterial cell is detectable as early as 3 min after phage addition, and that a significant luminescent signal is retained even after the inhibition of transcription or translation. We further show that mixing such phages with an *E. coli* strain surrounded by a K5 capsule, that is, a non‐cognate host of the K1F phage does not allow the release of the fusion proteins from the capsid head, and therefore does not result in a luminescent signal.

## 2 METHODS AND MATERIALS

### 2.1 Strains, chemicals and media

*E. coli* MDS42^17^ (no RRID, NCBI Taxonomy ID: 1110693, engineered in our laboratory earlier) was used for plasmid assembly and cloning, and *E. coli* BL21(DE3) (no RRID, NCBI Taxonomy ID: 469008, a kind gift of Frederick R. Blattner) was applied to make purified CnLuc protein extracts. Phage K1F was propagated on *E. coli* EV36 (no RRID, no NCBI Taxonomy ID) which is a K12/K1 hybrid developed by conjugation of Hfr kps + strain^18^ (both kindly provided by Dr. Eric R. Vimr). Bacterial cultures were grown in lysogeny broth (LB).^19^ Phage dilutions were prepared in buffer Φ80+ containing 0.1 M NaCl, 0.01 M Tris (pH 7.9), 0.01 M CaCl_2_ and 0.01 M MgCl_2_.^20^ To make soft agarose for phage titering and plaque assays, LB‐agar plates were overlain with 0.5% Seakem LE agarose (Lonza, Basel, Switzerland) containing 5 mM CaCl_2_ and 5 mM MgCl_2_. Antibiotics and inducers were ordered from Sigma‐Aldrich (St. Louis, MO, USA), and were used in the following concentrations: ampicillin (Ap): 50 μg/mL, chloramphenicol (Cm): 25 μg/mL, rifampicin: 50 μg/mL, tetracycline (Tc): 12.5 μg/mL, IPTG: 0.5 mM, anhydrotetracycline (aTc): 175 ng/mL. *E. coli* trains MDS42, BL21(DE3) and EV36, as well as phage K1F have all been validated previously, and have never before been reported as misidentified or contaminated. Each line was free of mycoplasma infection during the experiments described below.

### 2.2 Plasmid construction

The donor plasmids pSBC3g6.7::NnLuc and pSBC3g14::NnLuc, used for phage engineering, were constructed using circular polymerase extension cloning (CPEC)^21^ by fusing three PCR fragments. The three fragments were obtained by amplifying (i) an older donor plasmid, pSBC3,^22^ (ii) the g6.7 or g14 gene of K1F, and (iii) the NnLuc gene. The NnLuc gene was ordered as GeneStrands from Eurofins Genomics (Ebersberg, Germany). Details of the assembly are described in the Supplementary Methods.

The pET24CnLuc_his plasmid was constructed by CPEC by fusing two PCR fragments. These fragments were obtained by amplifying: (i) the backbone of the pET24dihyd_his_tetR expression plasmid and (ii) the CnLuc gene, ordered as a GeneBlock from IDT (Leuven, Belgium). The pZA31CnLuc_his_tetR plasmid was made by fusing two PCR fragments using CPEC. These fragments were obtained by amplifying: (i) the backbone of the pZA31CFPtetR plasmid,^23^ and (ii) the CnLuc gene, along with its RBS, hexahistidine tag, and stop codon from pET24CnLuc_his. The construction of both expression plasmids is detailed in the Supplementary Methods.

### 2.3 Bacteriophage engineering

Phages K1Fe6.7::NnLuc and K1Fe14::NnLuc were generated using plasmid‐mediated phage genome editing.^24^, ^25^ The prior phage carries an extra copy of gene g6.7 in translational fusion with the N terminal part of nanoluciferase (g6.7::NnLuc), integrated into the phage genome just downstream of gene g10b. The latter phage carries an extra copy of gene g14 in translational fusion with the N terminal part of nanoluciferase (g14::NnLuc), integrated into the phage genome just downstream of gene g10b. For the editing, phage K1F was grown on *E. coli* EV36 containing the donor plasmid pSBC3g6.7::NnLuc or pSBC3g14::NnLuc, respectively. The resulting phage mixes were grown on EV36 + pCas9K1FC2 (described in^22^) for three rounds of phage growth. The third phage lysate was diluted to obtain isolated plaques on a lawn of *E. coli* EV36, and the plaques were screened by plaque PCR, using primer pairs g10fw + NnLucCheckRev and NnLucCheckFw + g11rev. Both checking PCRs were done using Taq polymerase at 57°C annealing temperature.

Basic processes, such as phage growth, plaque assay, titering, and phage recovery from plaques were done using established protocols.^26^

### 2.4 Preparation of purified CnLuc

Suspensions of purified CnLuc protein were made by transforming BL21(DE3) cells with the pET24CnLuc_His plasmid. An overnight starter culture, generated from a single colony of the obtained cell line was diluted 100x in 50 mL of LB + Ap medium. After 1.5 h of growth at 37°C, IPTG was added, and cells were harvested after a further 3 h of growth. Cells were pelleted at 10.000 g for 10 min and resuspended in 1 mL of equilibration buffer (20 mM Na_2_HPO_4_, 300 mM NaCl, 10 mM imidazole, pH 7.4). Next, cells were lysed by sonication for 4 rounds, 30 s each, with 30 s interruptions, followed by pelleting at 10.000 g for 10 min. Finally, the supernatant was passed through a 1 mL His‐Pur Ni‐NTA Spin Column (Thermo Fisher Scientific, Waltham, MA, USA) and eluted using the kit's elution buffer following manufacturer's instructions.

### 2.5 Luminescence measurements

In order to measure luminescence, we mixed 100 μL of various samples with 100 μL of prepared NanoGlo reagent (buffer + substrate) in a white flat bottom 96‐well plate obtained from Thermo scientific. Samples were then instantly (<30 s) examined in a Synergy H1 reader using blue luminescence fibers (460 nm). Gain was set to 140. The Gen5 3.11 program was used for instrument control and data collection. To minimize signal crosstalk between neighboring wells, samples were laid out in the microplates with one “isolator well,”, containing 200 μL of sterile LB medium, separating each group of parallels.

Cellular samples were prepared by growing *E. coli* EV36 cultures (optionally harboring pZA31CnLuc_his_tetR) induced with aTc, to an OD600 of 0.6 in liquid LB medium, from which they were pipetted into appropriate wells, 35 μL each. Additionally, phage lysates were pipetted into the sample wells, 65 μL each, near‐simultaneously with a multichannel pipette, totaling the volume to 100 μL/sample.

For the kinetic measurements, the timer was started when the phages were added to the samples. The NanoGlo reagent was added to the sample wells in a kinetic series followed by a 10 s shaking and luminescence measurement. Each kinetic time point represents the start of the actual luminescence measurement. NanoGlo addition and thus the chemical lysis of the cells occurred approximately 30 s before each kinetic time point.

For the experiments analyzing cell‐free CnLuc, an overnight culture of BL21(DE3) (optionally harboring pZA31CnLuc_his_tetR plasmid) was diluted 100‐fold and grown for 4 h at 37°C in 5 mL of liquid LB medium + aTc. Cells were pelleted at 10.000 g for 1 min and resuspended in 0.5 mL equilibration buffer (see above). Cells were lysed by 4 rounds of sonication, lasting 30 s each, with 30 s pauses in between, then centrifuged for 5 min at 10.000 g. The supernatant was collected for testing CnLuc activity.

To analyze heat‐treated phages, bacteriophages were incubated at 75°C for 5 min. Next, 300 μL of heat‐treated or untreated phages were mixed with 75 μL of the sonicated cell supernatant. The mixture was left at room temperature for 10 min and divided into wells, 100 μL each, followed by luminescence measurement.

For the model diagnostic system, *E. coli* EV36 cultures were grown to an OD600 of 0.6 in liquid LB medium. The *E. coli* Nissle1917 strain was used as a negative control. 30 μL of the bacterial cultures was challenged with 30 μL of either K1Fe6.7::NnLuc or K1Fe14::NnLuc phage suspension. The reaction mixture was supplemented with 40 μL of column‐purified CnLuc solution to add up to a final 100 μL volume. The reaction was stopped after 3 or 12 min by the addition of the NanoGlo reagent, and luminescence was measured after 10 min of incubation.

## 3 RESULTS

To generate K1Fe6.7::NnLuc phages, which carry an extra copy of gene g6.7 in translational fusion with the N terminal part of nanoLuciferase (NnLuc), we carried out the procedures described in Methods and Materials. In summary, wild type K1F phages were grown on *E. coli* EV36 harboring the donor plasmid (pSBC3g6.7::NnLuc). To select for recombinant phages, the resulting phage mix was grown on EV36 + pCas9K1FC2, which cleaves the wild type, but not the recombinant phage's genome. Phage K1Fe14::NnLuc was generated in a similar fashion, using the donor plasmid pSBC3g14::NnLuc. Phenotypic testing of the recombinant phages revealed that they yield similar titres as the wild type, with K1Fe14::NnLuc displaying a slight reduction in plaque size (Figures S1–S3 in the Supplementary Results).

### 3.1 In vitro tests

#### 3.1.1 Testing the activity of NnLuc fusion proteins released from phage heads

**FIGURE 1:**
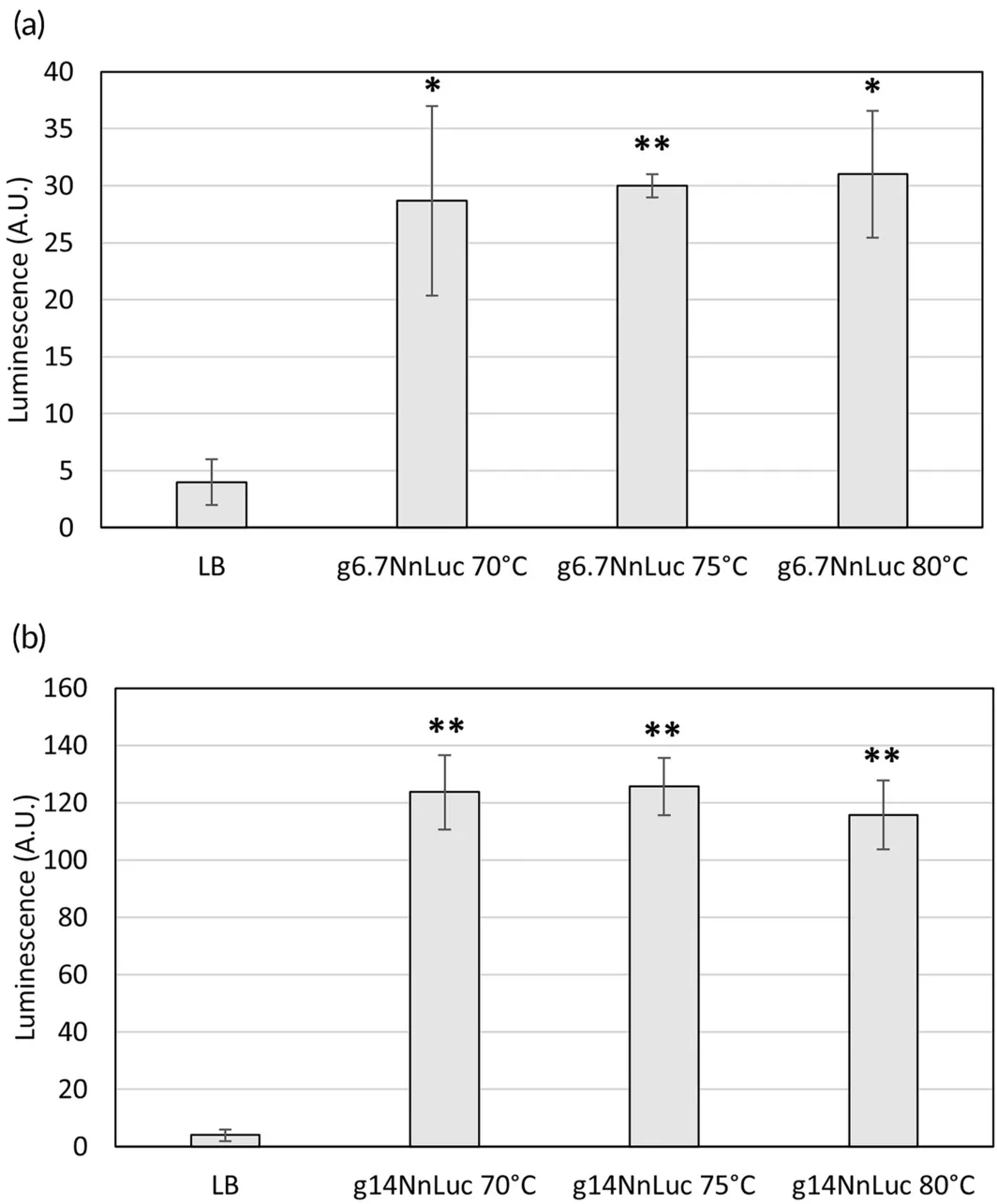
Activity of NnLuc fusion proteins released from the heads of heat‐treated phages. K1Fe6.7::NnLuc (a) and K1Fe14::NnLuc (b) phages were heat‐treated at the indicated temperatures for 5 min, and mixed with CnLuc extracted from producer cells, as described in the methods. Means of 3 parallel measurements are shown, made on independent samples. Error bars mark standard deviation (SD). Asterisks mark the results of two‐tailed, unpaired *t*‐tests comparing each value to the background signal (LB). *: *p*< 0.01; **: *p*< 0.0001.

The genomes of K1Fe6.7::NnLuc and K1Fe14::NnLuc phages contain an extra copy of the gene g6.7 and g14, respectively, in translational fusion with the NnLuc gene. To test whether these fusion genes (g6.7::NnLuc and g14::NnLuc) are packaged into the phage heads, we released all capsid‐enclosed proteins by heat inactivating the recombinant phages. These heat‐inactivated phages were mixed with the cellular extract of *E. coli* BL21(DE3) harboring the induced pZA31CnLuc_his_tetR plasmid. The binding of g6.7::NnLuc and g14::NnLuc, potentially released from the phage heads to the CnLuc proteins extracted from the induced producer *E. coli* should give a luminescence signal measurable at 460 nm. Indeed, the luminescence values were significantly above the background levels measured for empty LB medium (Figure 1).

**FIGURE 2:**
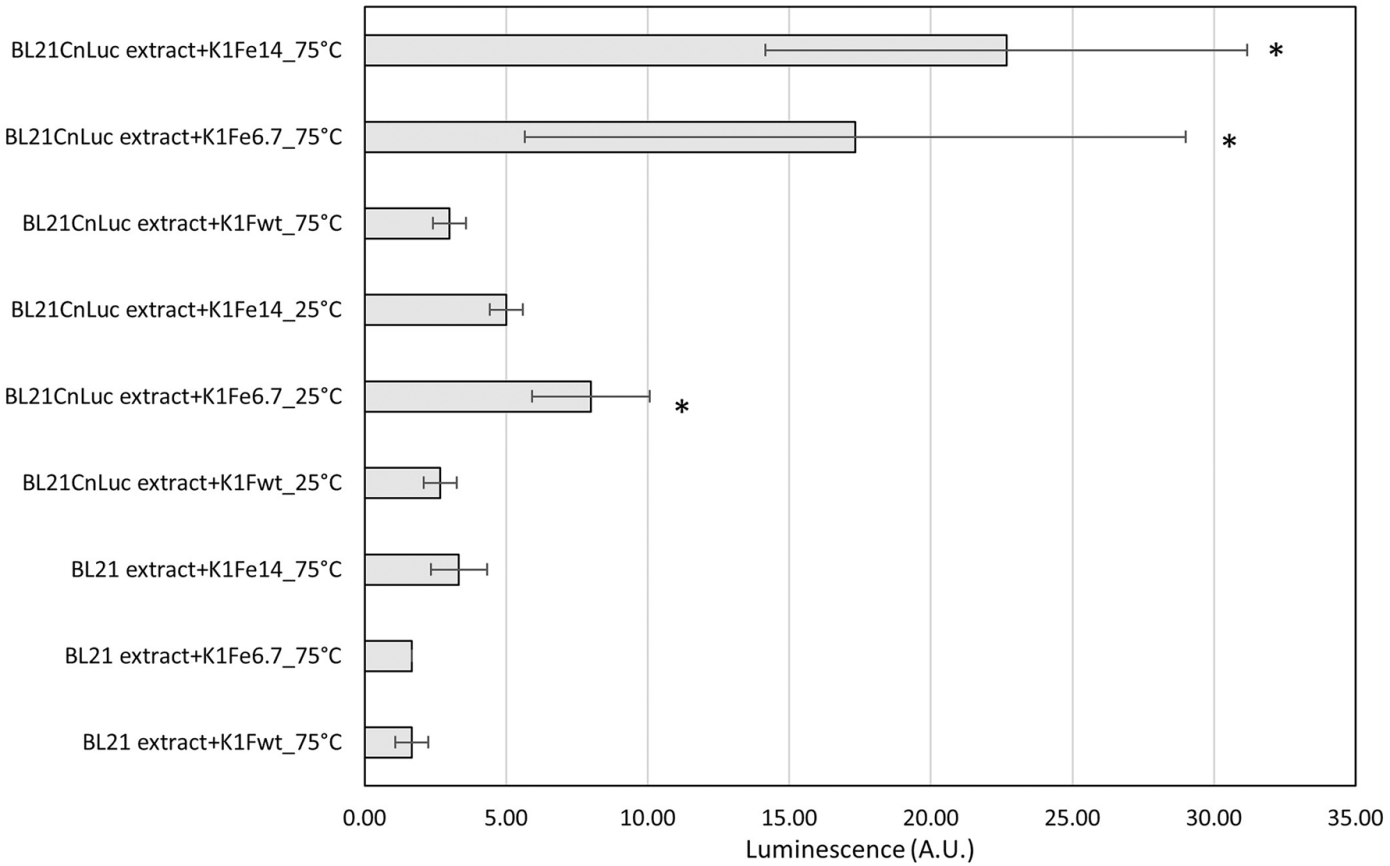
Activity of NnLuc fusion proteins released from the heads of heat‐treated phages. BL21CnLuc marks extracts of *Escherichia coli*BL21(DE3) cells harboring induced pZA31CnLuc_his_tetR plasmid, BL21 marks plasmidless *E. coli* BL21(DE3) cells, K1Fe6.7 marks K1Fe6.7::NnLuc phages and K1Fe14 marks K1Fe14::NnLuc phages. Means of 3 parallel measurements are shown, made on independent samples. Error bars mark SD. Asterisks mark the results of two‐tailed, unpaired t tests comparing each value to the control (BL21(DE3) + K1Fwt) signal. *: *p*< 0.05.

In a second experiment, the luminescence signals deriving from similar samples (cell extract + phages) were compared to those produced by the control: plasmidless BL21(DE3) extract mixed with heat‐treated wild type (wt) K1F phage. Heat treated K1Fe6.7::NnLuc and K1Fe14::NnLuc phages produced luminescence signals that were significantly higher than the control when mixed with the extract of BL21(DE3) + induced pZA31CnLuc_his_tetR (Figure 2). No increase in signal was observed when the extract derived from plasmidless cells. Similarly, no increase in luminescence was seen when the BL21(DE3) + induced pZA31CnLuc_his_tetR extract was mixed with heat‐inactivated K1F wt phage or intact (non‐heat treated) K1Fe14::NnLuc phage. The only unexpected increase in luminescence was seen when BL21(DE3) + induced pZA31CnLuc_his_tetR extract was mixed with intact K1Fe6.7::NnLuc phage, probably caused by contaminating soluble g6.7::NnLuc. We concluded that fusion proteins g6.7::NnLuc and g14::NnLuc are packaged into K1Fe6.7::NnLuc and K1Fe14::NnLuc phages, respectively, and these recombinant proteins are capable of producing light when liberated and bound to free CnLuc.

### 3.2 In vivo experiments

#### 3.2.1 Endpoint luminescence measurements of phage‐bacterial interactions

**FIGURE 3:**
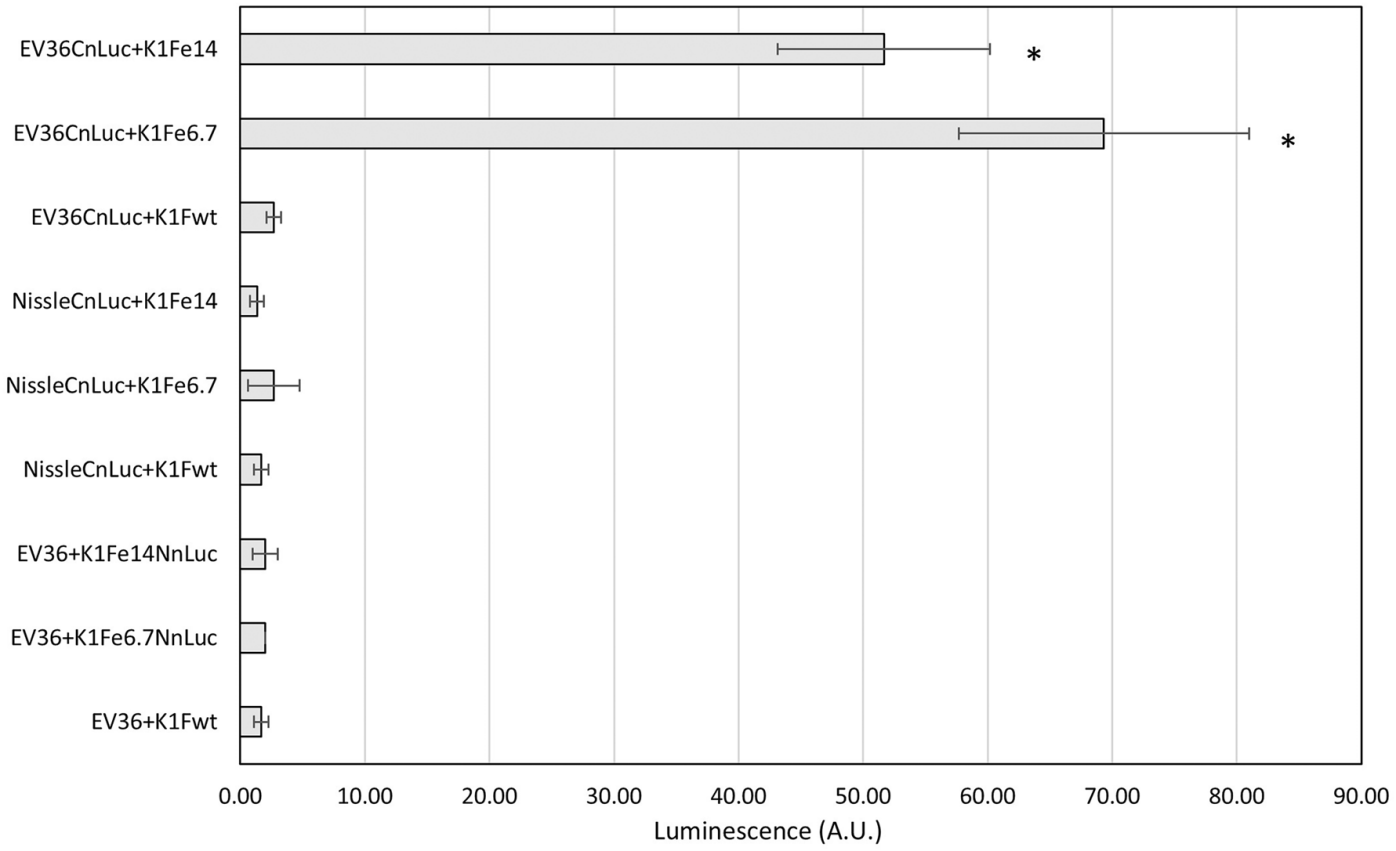
Endpoint luminescence signals produced by various phage‐bacterial interactions. EV36CnLuc marks *Escherichia coli*EV36 cells harboring induced pZA31CnLuc_his_tetR plasmid, EV36 marks plasmidless *E. coli* EV36 cells, NissleCnLuc marks *E. coli* Nissle1917 cells harboring induced pZA31CnLuc_his_tetR plasmid. Means of 3 parallel measurements are shown, made on independent samples. Error bars mark SD. Asterisks mark the results of two‐tailed, unpaired *t*‐tests comparing each value to the negative control (plasmidless *E. coli* EV36 + K1F wt phage). *: *p*< 0.001.

In these experiments, bacteria expressing the CnLuc gene from a plasmid were challenged with recombinant phages K1Fe6.7::NnLuc and K1Fe14::NnLuc. The negative control was the plasmidless *E. coli* EV36, challenged with the K1F wt phage. The luminescence signals measured ~12 min after the addition of the phages were significantly higher than the negative control in the case of *E. coli* EV36 + pZA31CnLuc_his_tetR when challenged by either recombinant phage (Figure 3). The luminescence was indistinguishable from the control in the case of *E. coli* Nissle1917 + pZA31CnLuc_his_tetR challenged with either phage, or plasmidless EV36 challenged with either phage. This is the expected result, for the cognate host (*E. coli* EV36, covered by the K1 capsule and carrying the induced pZA31CnLuc_his_tetR) binds to the phage and allows the injection of the phage capsid content leading to the binding of CnLuc to the injected NnLuc fusion protein, ultimately producing a luminescence signal. (In this case the NnLuc fusion protein derives both from the virion content and from the transcription/translation of the injected phage DNA.) Also as expected, the non‐cognate host (*E. coli* Nissle1917, covered by the K5 capsule and carrying the induced pZA31CnLuc_his_tetR) produced no luminescence signal with either recombinant phage, since binding of the phages to it is not possible, precluding the injection of the capsid content. In the other samples, the N or the C terminal fragment of the split nanoluciferase was missing, explaining the lack of luminescence.

#### 3.2.2 Kinetic luminescence measurements of phage‐bacterial interactions

**FIGURE 4:**
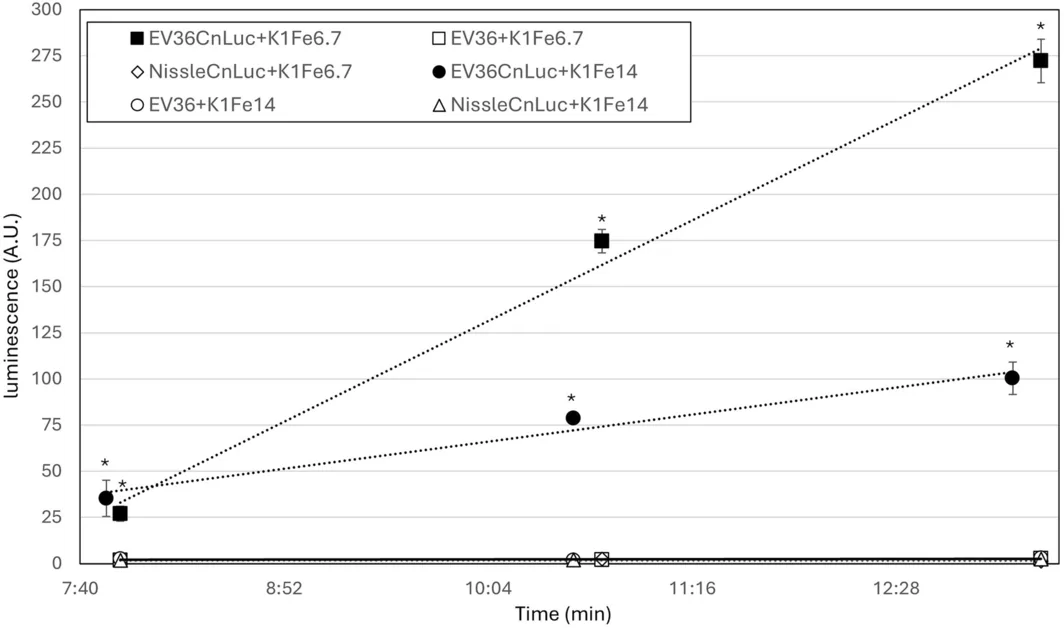
Kinetics of the luminescence signal produced by various phage‐bacterial interactions. *Escherichia coli*EV36 + induced pZA31CnLuc_his_tetR (EV36CnLuc) or *E. coli* Nissle1917 + induced pZA31CnLuc_his_tetR (NissleCnLuc) were challenged with K1Fe6.7::NnLuc or K1Fe14::NnLuc phages. The reaction was stopped by the addition of the NanoGlo reagent at the time indicated, and the luminescence was recorded. Means of 3 parallel measurements are shown, made on independent samples. Error bars mark SD. Asterisks mark the results of two‐tailed, unpaired t tests comparing each value to the negative control (plasmidless *E. coli* EV36 + the respective phage). *: *p*< 0.01.

In these experiments, bacteria expressing CnLuc from a plasmid were challenged with recombinant phages K1Fe6.7::NnLuc and K1Fe14::NnLuc. The reaction was stopped by addition of the NanoGlo reagent at regular time intervals, and the resulting luminescence was recorded. We expected the cognate host (*E. coli* EV36, covered by the K1 capsule and carrying the induced pZA31CnLuc_his_tetR) to bind to the phage and allow the injection of the capsid content leading to the binding of cellular CnLuc to the injected NnLuc fusion protein, ultimately producing a luminescence signal. (Before 8 min, the NnLuc fusion protein can derive only from the injected virion content. After 8 min, additional NnLuc fusion protein is produced by the transcription/translation of the injected phage DNA^27^). We also expected the non‐cognate host (*E. coli* Nissle1917, covered by the K5 capsule and carrying the induced pZA31CnLuc_his_tetR) to not bind these phages, precluding the injection of the capsid content and therefore producing no luminescence signal. The two negative controls were the cognate host (*E. coli* EV36, plasmidless) challenged with the recombinant phage K1Fe6.7::NnLuc and K1Fe14::NnLuc, respectively. The luminescence signals measured ~7 min 50 s after the addition of either of the recombinant phages were significantly higher than the negative controls in the case of *E. coli* EV36 + pZA31CnLuc_his_tetR but were indistinguishable from the controls in the case of *E. coli* Nissle1917 + pZA31CnLuc_his_tetR (Figure 4). Since in the T7 bacteriophage family the transcription/translation of late phage genes starts at 8 min or later,^27^ the significantly increased luminescence signal measured at 7:50–7:55 mark the functionality of the NnLuc fusion proteins injected from the phage head.

#### 3.2.3 Verifying the source of luminescence

To further confirm that luminescence signals, significantly differing for the cognate and non‐cognate hosts, are produced by the injected NnLuc fusion proteins as opposed to being transcription/translation products of the injected phage genome, three experiments were made. The first one repeated the in vivo kinetic measurements but included luminescence signal detection at earlier time points. The second and third ones applied the transcription inhibitor rifampicin and the translation inhibitor Tc, respectively, to eliminate NnLuc synthesized by the target cell.

**FIGURE 5:**
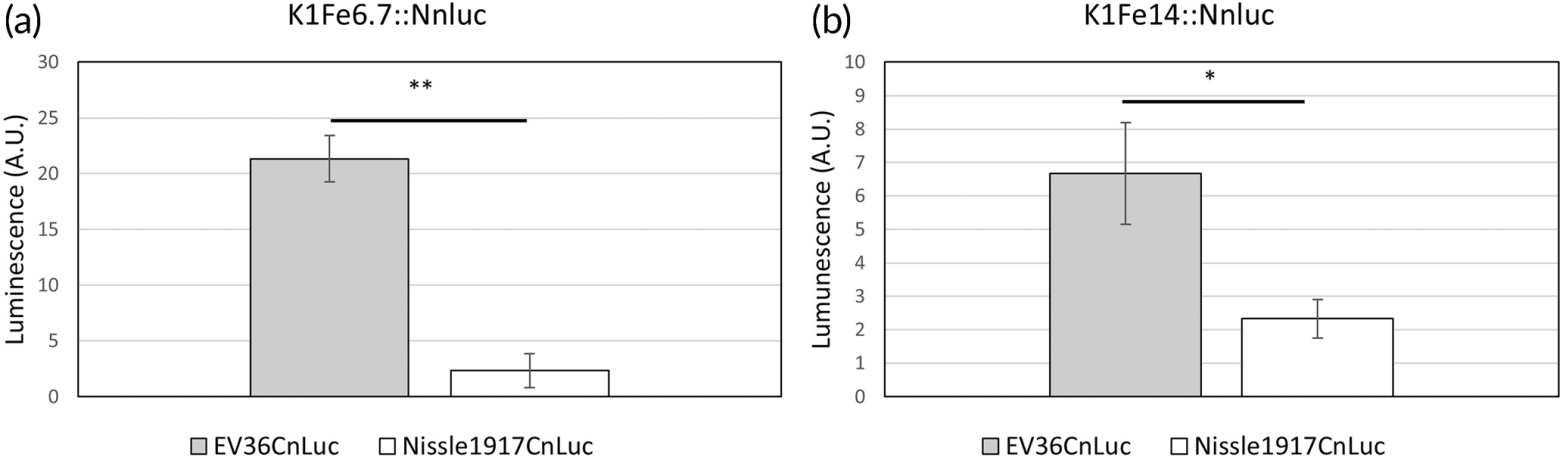
Verification of the functionality of injected NnLuc fusion proteins. Luminescence values were detected 5 min 45 s after challenging *E. coli* EV36 + induced pZA31CnLuc_his_tetR or *E. coli* Nissle1917 + induced pZA31CnLuc_his_tetR with either phage K1Fe6.7::NnLuc (a) or K1Fe14::NnLuc (b). Means of 3 parallel measurements are shown, made on independent samples. Error bars mark the SD. Asterisks indicate the results of two‐tailed, unpaired t tests comparing the values obtained with the two bacterial strains to each other. *: *p*< 0.05; **: *p*< 0.001.

Firstly, *E. coli* EV36 + induced pZA31CnLuc_his_tetR or *E. coli* Nissle1917 + induced pZA31CnLuc_his_tetR was challenged with either K1Fe6.7::NnLuc or K1Fe14::NnLuc phages, concentrated to a titre of 5 × 10^10^ phages/ml. After 5 min 45 s of incubation, NanoGlo buffer was added to lyse the cells, thereby functionally halting protein expression. After a further 10 min incubation, the NanoGlo substrate was added and the luminescence was recorded at 460 nm. As apparent on Figure 5, the luminescence signal detectable upon K1Fe6.7::NnLuc challenge (part A) or K1Fe14::NnLuc challenge (part B) was significantly higher when a cognate host (*E. coli* EV36) was coincident than in the presence of a non‐cognate host (*E. coli* Nissle1917). Since in the T7 bacteriophage family, the transcription/translation of late phage genes (including g6.7 and g14 orthologues) starts at 8 min or later,^27^ the significant luminescence signal measured at 5:45 provides further evidence for the functionality of the NnLuc fusion proteins injected from the phage head.

**FIGURE 6:**
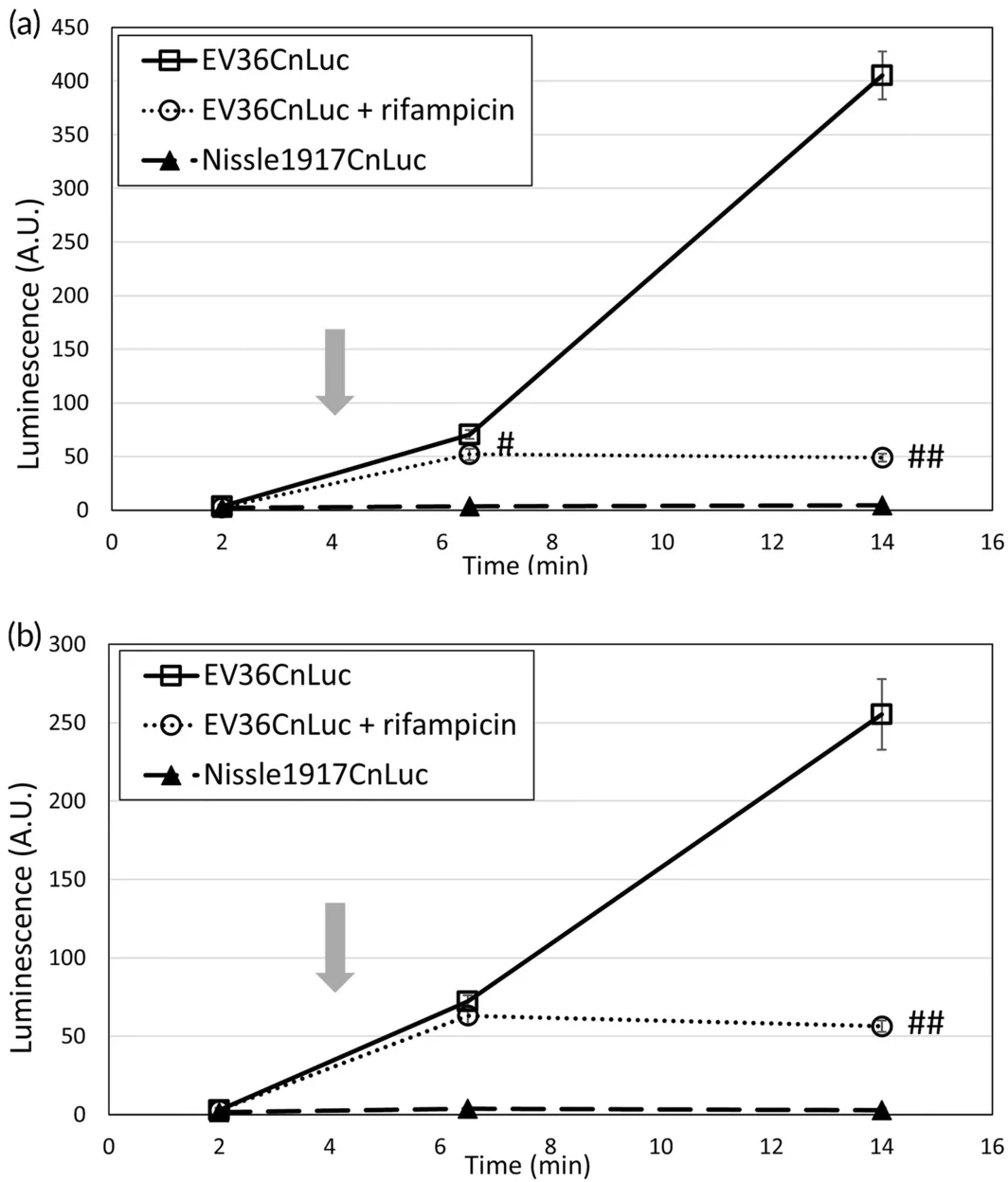
Effect of transcription inhibition on luminescence. Luminescence values were detected at various time points after infecting *Escherichia coli* EV36 + induced pZA31CnLuc_his_tetR (solid line) or *E. coli* Nissle1917 + induced pZA31CnLuc_his_tetR (dashed line) with either phage K1Fe6.7::NnLuc (a) or K1Fe14::NnLuc (b). Rifampicin was added at 4 min (gray arrow) to *E. coli* EV36 + induced pZA31CnLuc_his_tetR (dotted line). Means of 3 parallel measurements are shown, made on independent samples. Error bars mark the SD. Pound signs indicate the results of two‐tailed, unpaired t tests comparing the values of rifampicin‐treated cells to their untreated counterparts. #: *p*≤ 0.05; ##: *p*≤ 0.005.

In the second experiment, cognate (*E. coli* EV36 + induced pZA31CnLuc_his_tetR) or non‐cognate (*E. coli* Nissle1917 + induced pZA31CnLuc_his_tetR) host cells were challenged with either of the two recombinant phages (K1Fe6.7::NnLuc or K1Fe14::NnLuc). After 4 min of incubation, transcription in the cognate host was stopped by adding 50 μg/mL rifampicin (Figure 6). Rifampicin treatment had only a minor effect on the luminescence produced by the cognate host at 6 min 30 s: transcription inhibition caused a 26% decrease in the case of K1Fe6.7::NnLuc (*p* = 0.01) (Figure 6a), but there was no significant decrease resulting from transcription inhibition during K1Fe14::NnLuc infection (*p* = 0.089) (Figure 6b). Both phages could therefore produce luminescence upon infection of the target cell independently from RNA synthesis, implying that the signal derives from the injection of ready‐made NnLuc. As expected, the luminescence levels plateaued upon transcription inhibition, while a continuing increase was seen for the untreated cognate host, most likely originating from cell‐derived NnLuc expression.

**FIGURE 7:**
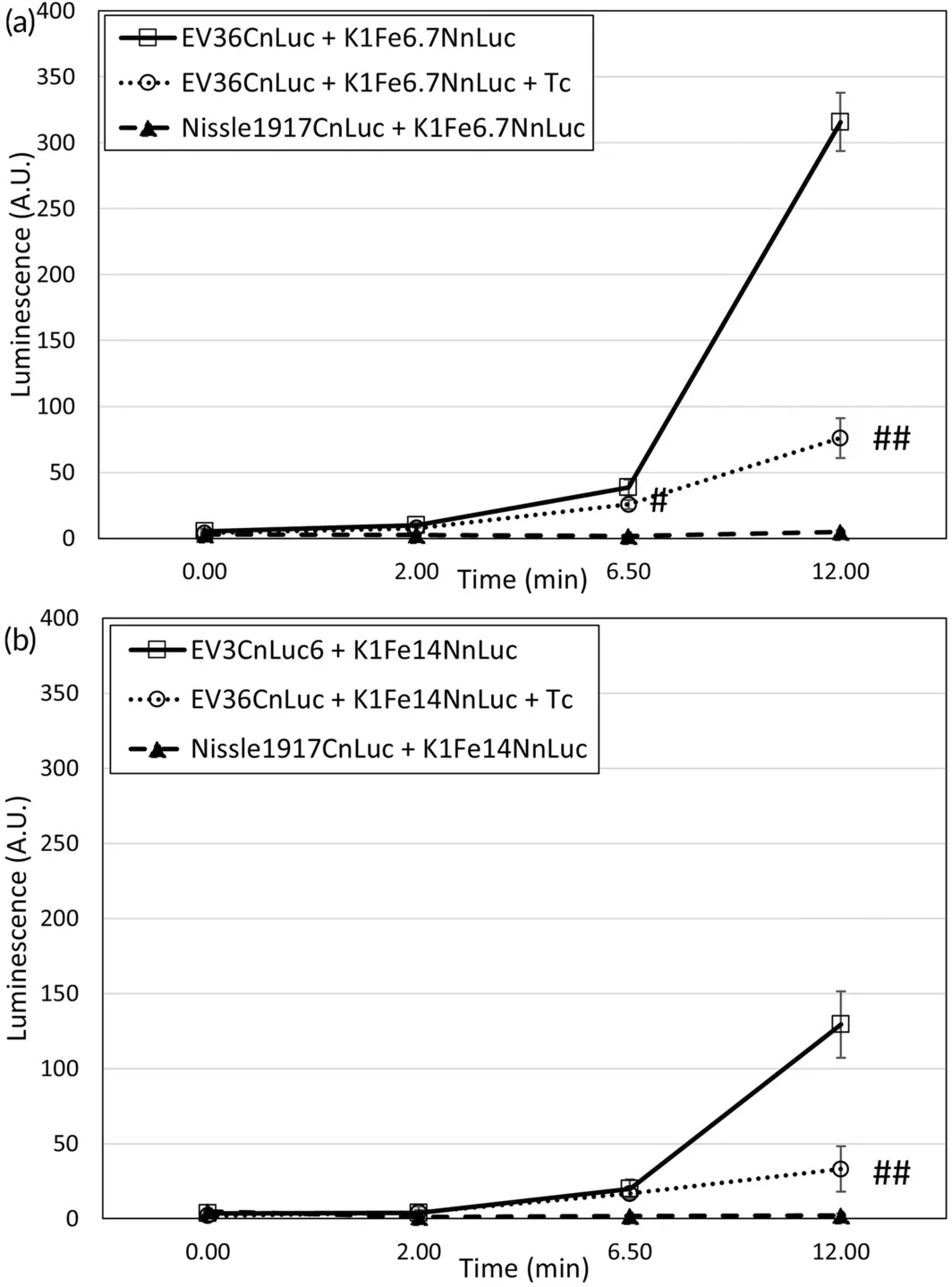
The effect of translation inhibition on the kinetics of luminescence during K1Fe6.7::NnLuc (a) or K1Fe14::NnLuc (b) infection. *Escherichia coli* EV36 + induced pZA31CnLuc_his_tetR (EV36CnLuc) or *E. coli* Nissle1917 + induced pZA31CnLuc_his_tetR (NissleCnLuc) were challenged with K1Fe6.7::NnLuc (a) or K1Fe14::NnLuc (b) phages. The reactions marked with “+ Tc” were treated with 12.5 μg/mL Tc 2 min before infection. Means of 3 parallel measurements are shown, made on independent samples. Error bars mark the SD. Pound signs indicate the results of two‐tailed, unpaired t tests comparing the values of Tc‐treated cells to their untreated counterparts. #: *p*≤ 0.05; ##: *p*≤ 0.0001.

In our third experiment interrogating the source of the luminescent signal, we inhibited cellular protein translation using tetracycline (Tc). CnLuc‐expressing *E. coli* EV36 cells were treated with Tc and infected with the recombinant phages (K1Fe6.7::NnLuc or K1Fe14::NnLuc) (Figure 7). Similarly to the rifampicin experiment, Tc had only a minor effect on the luminescence produced by the cognate host at 6 min 30 s: translation inhibition caused a 34% decrease in the case of K1Fe6.7::NnLuc (*p*< 0.05) (Figure 7a), but there was no significant decrease resulting from translation inhibition during K1Fe14::NnLuc infection (*p* = 0.28) (Figure 7b). The luminescence measured at 6 min 30 s was therefore predominantly attributable to protein injection for K1Fe6.7::NnLuc, and entirely in the case of K1Fe14::NnLuc. The luminescence increase continued for the untreated cells marking the continuation of cellular NnLuc synthesis. This rise was impeded but nevertheless continued in the case of the Tc‐treated cells, possibly indicating the incomplete inhibitory action of Tc on protein synthesis at short time frames. As a negative control, non‐cognate *E. coli* Nissle1917 cells were also included in the test but did not produce luminescence.

### 3.3 A model diagnostic system

**FIGURE 8:**
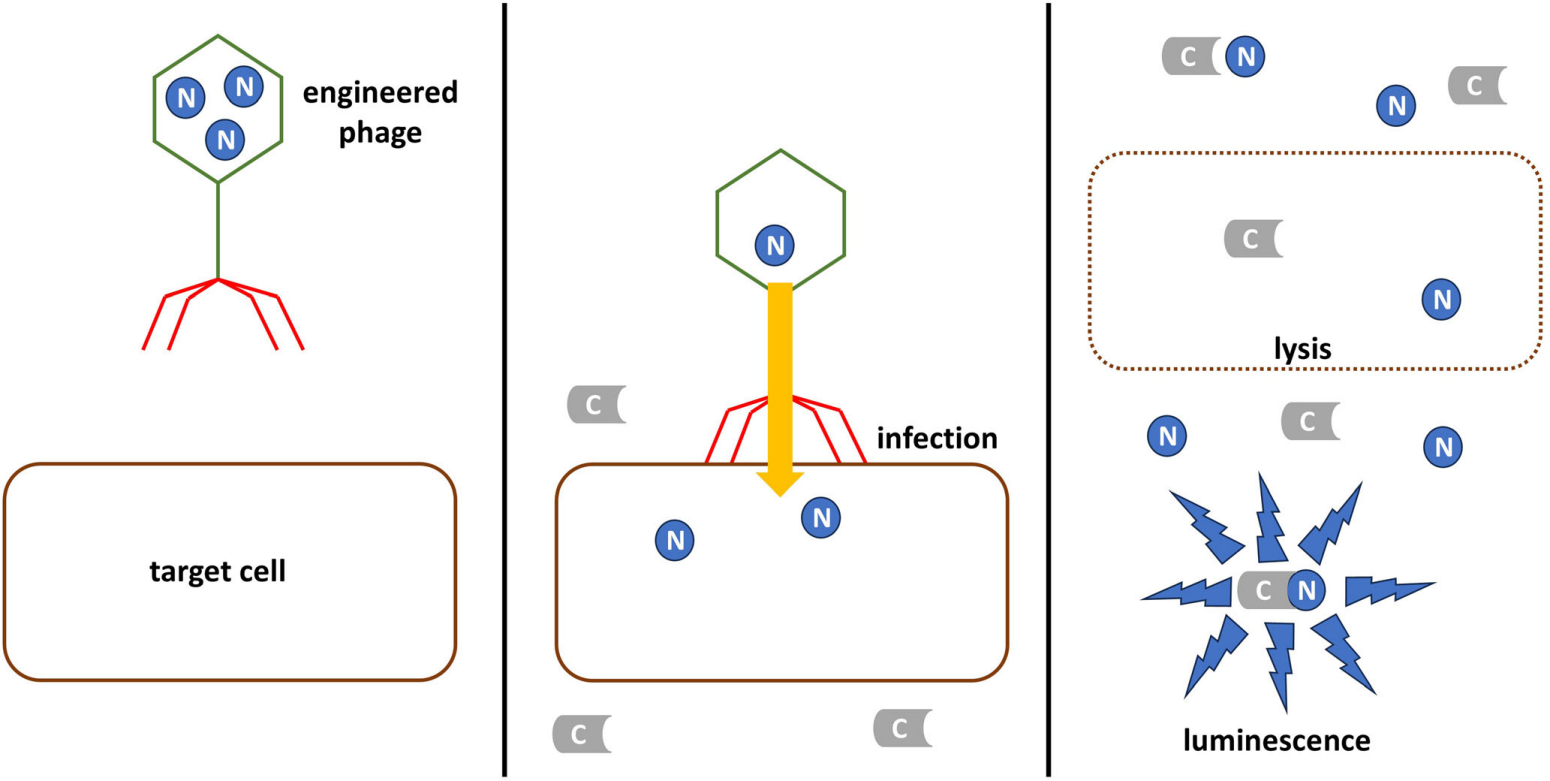
Schematic of the model diagnostic system. N and C represent the NnLuc and CnLuc protein fragments, respectively.

Up to this point, all in vivo experiments to generate luminescence upon phage infection required the expression of the CnLuc fragment by the target cell. Obviously, such systems are useless to detect naturally occurring bacterial strains due to the lack of CnLuc expression. To circumvent this problem, we designed a model diagnostic system where the cells are infected by the recombinant phages, followed by the extracellular administration of the CnLuc fragment, the substrate Furimazine and a cell lysis agent that allows their binding to the injected NnLuc fragment (Figure 8).

**FIGURE 9:**
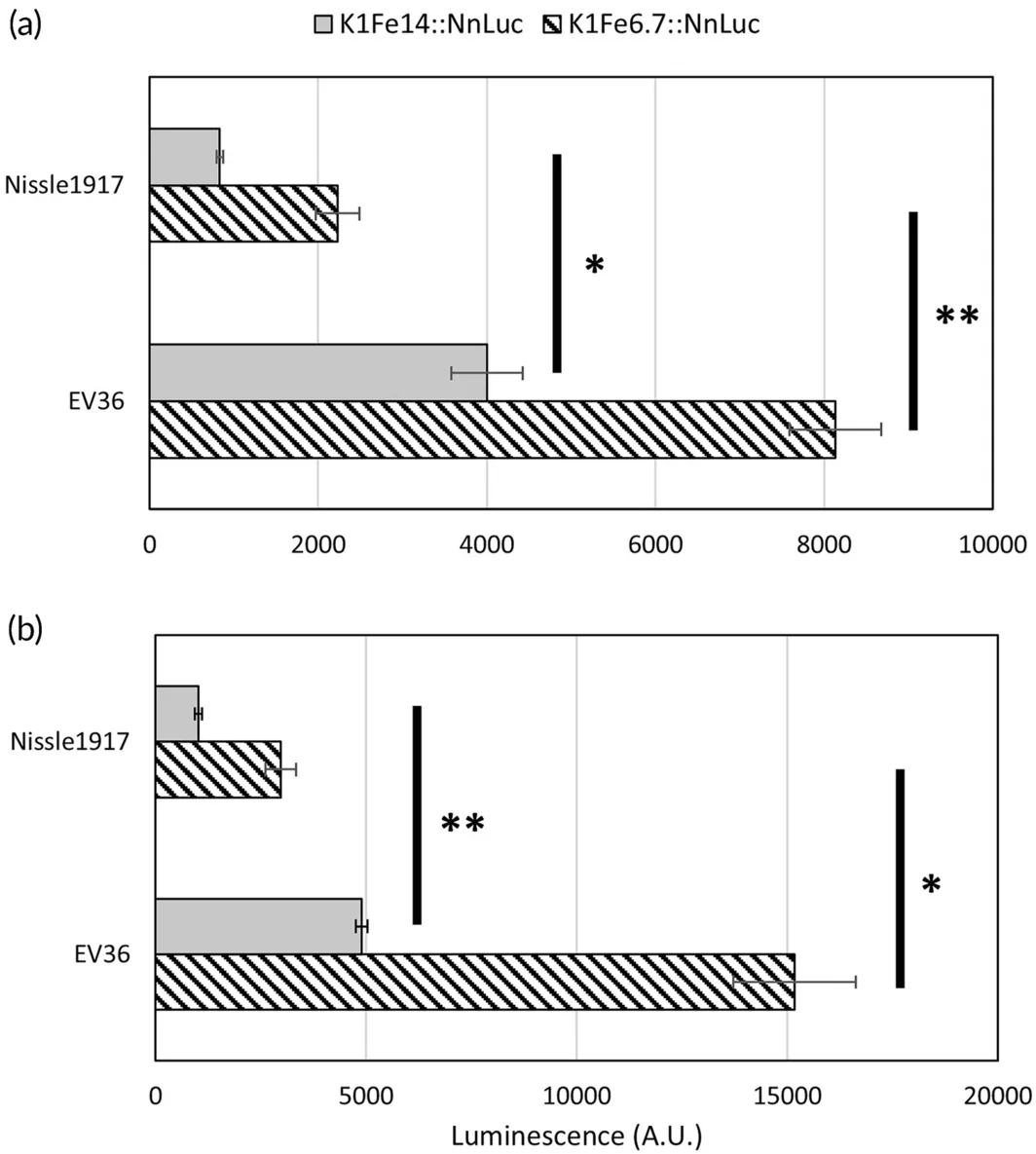
Luminescence output of the model diagnostic system. Plasmidless *Escherichia coli*EV36 or *E. coli* Nissle1917 cells were mixed with phage suspensions of K1Fe6.7::NnLuc (gray) or K1Fe14::NnLuc (diagonals), as well as with purified CnLuc protein, and the NanoGlo reagent was added 3 min (a) or 12 min (b) later, followed by luminescence readout. Means of 3 parallel measurements are shown, made on independent samples. Error bars mark the SD. Asterisks indicate the results of two‐tailed, unpaired t tests comparing the values generated by the two bacterial strains. *: *p*≤ 0.001; **: *p*≤ 0.0001.

The model diagnostic system was tested by challenging *E. coli* EV36 with either K1Fe6.7::NnLuc or K1Fe14::NnLuc phages. The *E. coli* Nissle1917 strain was used as a negative control. The reaction was stopped after 3 or 12 min by the addition of the NanoGlo reagent mixed with column‐purified CnLuc protein. The NanoGlo reagent contains the substrate Furimazine along with a proprietary cell lysis agent. As a result, the luminescence generated by the cognate host was significantly higher than that produced by the negative control at both timepoints for both phages (Figure 9). This experiment therefore validated the feasibility of using external CnLuc proteins for detecting wild‐type bacteria. These results also reinforced the selectivity of our phage‐based bacterial detection system, and the 3 min signal provided the strongest evidence yet that a robust, detectable luminescent signal can be generated solely by the injected proteins, before the appearance of cell‐derived NnLuc.

### 3.4 Determining the sensitivity of detection

**FIGURE 10:**
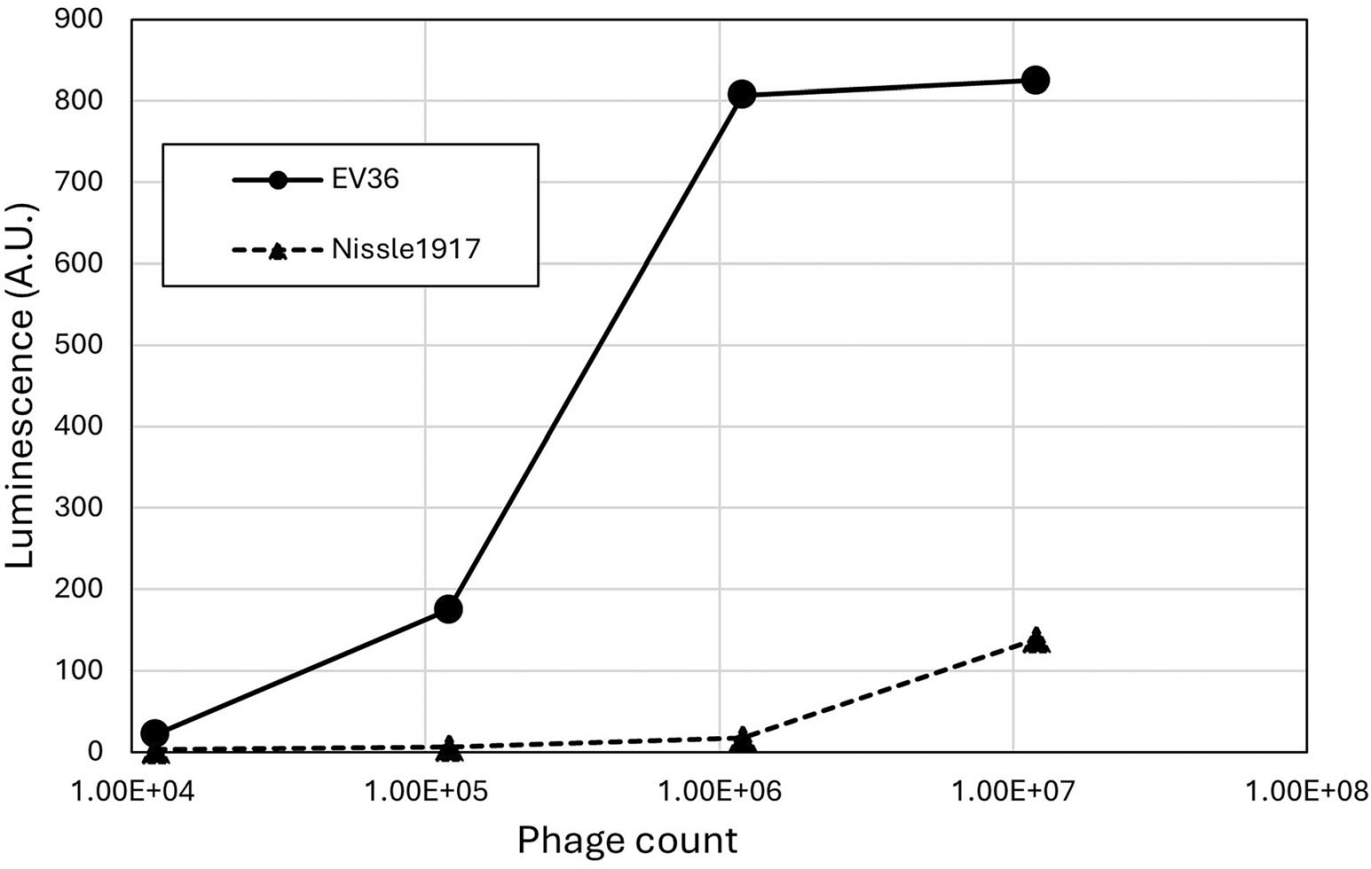
The dependence of the luminescent signal on phage count, produced by cognate (EV36, solid line) or non‐cognate (Nissle1917, dashed line) cells. The cells were challenged by K1Fe6.7::NnLuc, and CnLuc was provided extracellularly in the form of a purified suspension.

Besides the selectivity of our system, which was demonstrated by simultaneously challenging K1 and K5 capsule‐expressing *E. coli* strains, it is also of great importance to know the sensitivity of detection. In practice, this means the lower limit of cell count that can generate a signal significantly higher than the negative control. Prior to the actual measurements to determine the limit of detection, we first carried out a pilot study aiming to reduce the noise. This seemed necessary since the reaction harboring the negative control cell line, Nissle1917 produces a signal that is apparently higher than zero (Figure 9). We found that this noise could be diminished by reducing the phage count: when using 1.2 × 10^5^ phages in the reaction, the luminescence of the negative control became indistinguishable from the background signal, while the signal of the cognate host EV36 was still 29‐fold higher (Figure 10).

**FIGURE 11:**
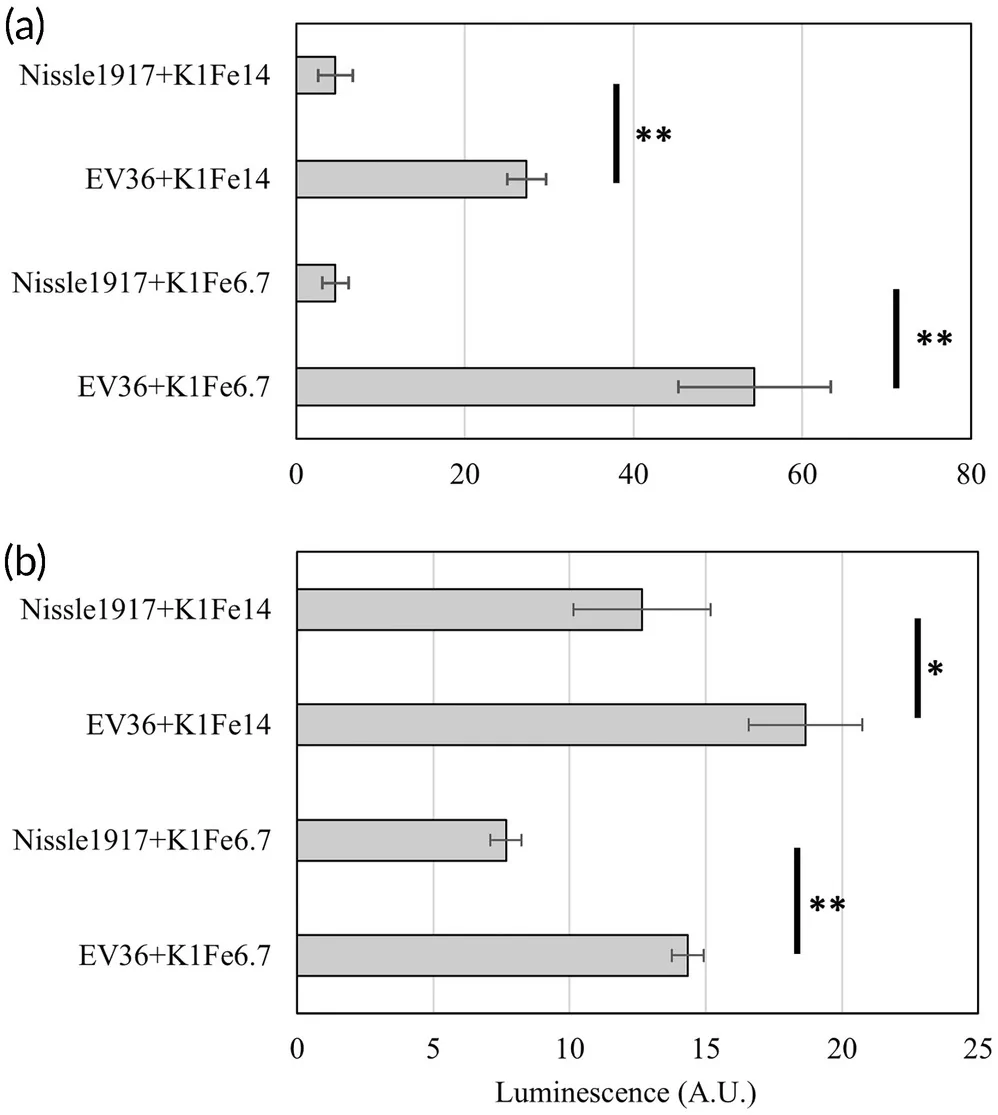
Luminescence values measured at the limit of detection. The charts display the significant difference of the luminescence signals emitted by positive (EV36) and negative (Nissle1917) samples measured after an 8 min (a) or a 15 min (b) phage infection. The cells were challenged with K1Fe6.7::NnLuc or K1Fe14::NnLuc phages, and CnLuc was provided extracellularly in the form of a purified suspension. Means of 3 parallel measurements are shown, made on independent samples. Error bars mark the SD. Asterisks indicate the results of two‐tailed, unpaired t tests comparing the values generated by the two bacterial strains. *: *p*≤ 0.05; **: *p*≤ 0.001.

Based on the above results, sensitivity tests of the model diagnostic system were carried out using phage counts of 1.2 × 10^5^ K1Fe6.7::NnLuc in each well of the microplate. Various dilutions of the cognate (EV36) or non‐cognate (Nissle1917) cells were added to each well, and the generated signals were compared for the two cell lines at corresponding dilutions, seeking the lowest cell count that generates a significant difference. We found that the signal intensity is dependent on the incubation time of both the phage infection and the luminescent reaction, with longer times resulting in stronger signals. Consequently, the timespans of these two reactions influence the measure of sensitivity. At short infection times (8 min), when the signal derived from injected fusion proteins only, the sensitivity of detection was 6.9 × 10^5^ cells/well, corresponding to a sample concentration of 2.3 × 10^7^ cells/mL (Figure 11a). If the cells were lysed after 15 min of phage infection, and NanoGlo reagent addition was followed by 35 min of incubation before readout, the limit of detection improved to 1.2 × 10^4^ cells/well, corresponding to a sample concentration of 3.5 × 10^5^ cells/mL (Figure 11b). We therefore found a trade‐off between the timespan of the diagnostic test and its sensitivity.

## 4 DISCUSSION

The aim of this work was to test whether injection of a protein from inside the capsid head of a phage into the cytoplasm of a cognate bacterium can be directly detected using readily available laboratory hardware. The general strategy was to fuse phage ICPs, destined to enter the target cell, to readily‐detectable protein reporter partners. In principle, the reporters could be chromogenic^28^ or fluorescent^29^ proteins. However, we decided to use the nanoluciferase protein as a fusion partner because luminescence is claimed to possess a signal‐to‐noise ratio that is far superior to the two prior reporter categories.^30^

Nanoluciferase is an engineered derivative of the luciferase of *O. gracilirostris*, which offers a small size (19.1 kDa), long activity half‐life and a high specific activity.^31^ A further refinement to our design was the use of the split, self‐assembling form (NnLuc with CnLuc) of nanoluciferase^32^ which circumvents two potential problems: background luminescence originating from unattached phages and concerns that larger fusions might disrupt ICP capsid entry/exit.

The main principle in our phage design was to minimize the fitness cost of fusing a reporter protein to an ICP. For this reason, we inserted an additional copy of the gene encoding the ICP::NnLuc engineered fusion, leaving intact the wild‐type ICP gene. Our fusion genes were driven by the natural promoter of the ICP and were inserted in a genomic position of the K1F phage that we found to withstand insertions without an observable genetic instability.^22^, ^33^ To provide some flexibility to our tests and as a first step to determining the general applicability of our approach, we conducted parallel experiments fusing the reporter protein to the two smallest ICPs: g6.7 and g14. Based on the same reasoning, we chose NnLuc, the smaller of the two split fragments to be encoded on the phage genome as the fusion partner.

Our initial, in vitro experiments applied heat treatment to release the content of the phage capsids in the presence of His‐purified recombinant CnLuc proteins. The emerging luminescence signals detected successfully verified the presence and the functionality of the ICP::NnLuc proteins in the capsid of both recombinant phages (K1Fe6.7::NnLuc and K1Fe14::NnLuc). Next, we carried out in vivo experiments that monitor luminescence when CnLuc‐expressing *E. coli* cells are challenged with the recombinant phages. A significant increase in luminescence was only observed if the CnLuc‐expressing *E. coli* strain was surrounded by the K1 capsule, known to be required for K1F docking. *E. coli* Nissle1917, expressing CnLuc, but covered by a K5 capsule, did not display a luminescence increase when challenged by either of the recombinant phages. This is a demonstration of the potential selectivity of the test, indicating that phage binding is a prerequisite of NnLuc injection, nLuc assembly, and subsequent luminescence. Most importantly, the detectable luminescence increase was already significant at 5 min 45 s for both recombinant phages when infecting the cognate host. Late genes (including g6.7 and g14) of the T7 phage (and presumably of the closely related K1F) are expressed earliest at 7–8 min after infection.^34^ This indicates that transcription/translation of the reporter genes was not necessary for their detection. This conclusion was further supported by our observations made with two inhibitors of gene expression. The luminescent signal generated by phage K1Fe14::NnLuc 6 min 30 s after infecting a cognate host was independent of either transcription inhibition with rifampicin or translation inhibition with Tc. Similarly, the majority of the luminescent signal generated by phage K1Fe6.7::NnLuc infection was maintained in the presence of either inhibitor. These findings indicated that the entire, or nearly the entire luminescent signal measured 6 min 30 s after infection is independent of cellular gene expression and is most likely attributable to the injection of the NnLuc protein fragment. To the best of our knowledge, these are the first pieces of evidence that the injection of a protein from the phage into the target cell can be used to detect successful phage infection.

The use of our engineered phages in real‐life demands that wild type bacterial cells, which obviously do not express CnLuc, could also be detected. We display the development of such a setup, which we refer to as a model diagnostic system (Figure 8). This system requires the addition of purified CnLuc protein extracellularly to the infected bacteria, and some means of disruption of the cell envelope to ensure its access to the NnLuc fragments injected by the engineered phages. The NanoGlo reaction buffer, containing an undisclosed cell lysis agent turned out to fulfill this function. The column‐purified CnLuc administered extracellularly not only permitted luminescence generation upon phage infection but led to signal intensities two orders of magnitude higher than those seen with intracellularly expressed CnLuc. This probably indicates that the pZA31 plasmid‐derived expression of CnLuc in *E. coli* EV36 led to limiting amounts of this enzyme fragment, but its pET24 plasmid‐derived expression in *E. coli* BL21(DE3), followed by column purification, allowed us to bypass this limitation. As a result of the much stronger luminescence, the signals of positive versus negative strains became significantly different after 3 min of phage infection, providing the strongest evidence yet that the emitted light observed arises due to protein injection (Figure 9).

Prior to determining the sensitivity of our model diagnostic system we optimized the phage count to be used in this setup. We chose a phage count of 1.2 × 10^5^/reaction, since treating the negative control *E. coli* Nissle1917 cells with such a number of K1Fe6.7::NnLuc produced a luminescent signal that was indistinguishable from that of the empty medium, while treating the cognate host *E. coli* EV36 cells with a similar phage number yielded a 29‐fold higher signal (Figure 10). This phage count was used to challenge *E. coli* Nissle1917 and EV36 strains in various cellular concentrations. Concerning the actual limit of detection of positive (EV36) bacterial cells, we found a trade‐off between the minimal detectable cell number and the time‐span of the phage infection. If a short phage infection of 8 min was used relying mostly on the signal generated by the injected enzyme fragments, 6.9 × 10^5^ cells/well were required to observe the significantly increased signal of EV36 versus that of Nissle1917 (Figure 11a). If the phage infection lasted 15 min, allowing the de novo expression of NnLuc from the phage genome, and the NanoGlo reaction was incubated for 35 further min, the two strains produced significantly different signals even at cell counts of 1.2 × 10^4^ cells/well (Figure 11b). These cell counts correspond to cell concentrations of 2.3 × 10^7^ cells/mL and 3.5 × 10^6^ cells/mL, respectively. Considering that in an earlier report, the mean concentration of *E. coli* K1 isolated from meningitis patients fell in‐between 2 × 10^4^ and 4 × 10^7^ cells/mL,^35^ with a mean of 2 × 10^5^ cells/mL, the model system (before any targeted optimization) already displays a level of sensitivity that falls within a clinically relevant range. After making further developments (discussed below) that improve the signal‐to‐noise ratio, and/or including a microcentrifuge‐based ultrafiltration step to concentrate the sample, the system could achieve the ability to conveniently span the bacterial concentration range expected in *E. coli* K1 meningitis.

This work establishes a proof of concept for the development of a diagnostic tool detecting specific bacterial strains against which NnLuc expressing phages are developed. This technology readily lends itself to cheap, lateral flow style diagnostic tests that allow for rapid detection in the field without need for any further device or sample cleanup prior to testing. Any diagnostic device would likely substitute a chromogenic reporter for ease of detection outside of a lab setting. Such a diagnostic would provide all elements required for signal generation other than the target (host bacteria). Taking the current work as an example, these would be Furimazine (the substrate of nLuc), lyophilized or otherwise dry‐stabilized CnLuc and of course the NnLuc‐bearing phage. The user would supply the host along with the moisture required to initiate phage‐host and enzyme‐substrate reactions. Potentially, a lysing reagent would also be added to facilitate union of cytosolic NnLuc with the supplied exogenous CnLuc.

The development of a reliable protocol is in progress. One shortcoming of our results is the false positive luminescent signal detected when mixing intact K1Fe6.7::NnLuc with a CnLuc extract (Figure 2). We believe this was caused by soluble NnLuc proteins present in phage lysates and underlines the importance of using high purity recombinant phages and recombinant CnLuc for detection. We provide an ultrafiltration protocol in the Supplementary Methods as a measure of reducing soluble NnLuc contamination in the phage lysates. Improved purity will likely reduce background luminescence and increase the signal‐to‐noise ratio of the system. That ratio may also be improved by deleting the intact copies of the wild‐type ICP genes from the genomes of the recombinant phages, thereby potentially elevating NnLuc quantity within each capsid and consequently increasing the achievable luminescent signal intensities. The fitness costs of phages purely expressing the labeled form of the ICP (g6.7::NnLuc or g14::NnLuc) need to be determined, however. If wild‐type ICP is found to be obligatory, a compromise may be to leave the wild‐type ICP copy but with an attenuated promoter, thereby enriching NnLuc capsid loading while maintaining viability. Signal intensities may also be increased by further concentrating phage suspensions by prolonging standard filter‐based purification.

Another approach could be to deploy this novel technology within a standard microfluidic device. Microfluidic lab‐on‐a‐chip designs, compared to their corresponding macroscopic reactions, have been shown to allow shorter reaction times and require less substrate– thus lowering the detection limit of the system. In many cases, the limited and strictly defined reaction spaces also permit higher sensitivity of detection and a further improvement in signal‐to‐noise ratio. We have displayed the schematic of a single‐channel microfluidic detection device elsewhere (Liyanagedera et al., submitted). The real power of microfluidics, however, lies in the possibility of fabricating multichannel devices, allowing the testing of multiple samples against multiple phages using combinatorial matrices. One can envision using multi‐step microfluidic diagnostic pipelines, where, for example, the first chip merely classifies the genus of the bacterial cells, the second chip (chosen based on the output of the first chip) provides information on the species, and the third chip (chosen based on the output of the second chip) defines the exact bacterial strain.

With the renaissance of phage therapy, defining the serotype of the pathogenic bacterial strain using such a phage‐based system far exceeds the value of mere classification: it provides direct, visually‐supported information on the phage sensitivity of the pathogenic isolate. The novel diagnostic phages presented in this work could be used in conjunction with therapeutic phages. By combining those phages that generate positive signals, phage cocktails can be quickly prepared and administered. This could result in the accelerated provisioning of personalized antibacterial therapies to previously unseen rates in precision medicine from hours to minutes – and in a context where minutes may matter when it comes to saving life and limb.

## 5 CONCLUSIONS

We have engineered phages that effectively package a nanoluciferase enzyme fragment into their capsids. The recombinant phages are capable of generating a luminescent signal in the presence of their cognate host and the missing fragment of nanoluciferase within an extremely short time (3 min). We have showcased a model diagnostic system which, using the engineered phages, can selectively detect the presence of the wild‐type cognate host strain at clinically relevant concentrations.

## AUTHOR CONTRIBUTIONS

**Ákos Avramucz:** Investigation; methodology. **Tamás Fehér:** Funding acquisition; supervision; writing – original draft. **Sahan B. W. Liyanagedera:** Writing – review and editing; visualization. **Richard Amaee:** Conceptualization; writing – review and editing; supervision; methodology. **Joseph P. Wheatley:** Investigation; methodology.

## FUNDING INFORMATION

TF was supported by the National Research, Development and Innovation Office (NKFIH) of Hungary (Biotechnology National Lab Grant No. 2022‐2.1.1‐NL‐2022‐00008) and by the NKFIH Advanced Grant (No. 152982).

## CONFLICT OF INTEREST STATEMENT

The authors intend to pursue commercialization of breakthrough via Lucidix Biolabs (LB). RA is Founder and Director at LB. TF and AA intend to pursue further work with LB toward commercial development. SBWL is co‐founder of Biophoundry who may work with LB toward commercial development. JPW may work with LB in the future toward commercial development.

## Supporting information


**Figure S1.** Comparison of phage titres obtained with various phages. The three phages were grown on *Escherichia coli* EV36 until complete lysis was observed. Values are means of three dilution replicates, error bars represent standard deviations.
**Figure S2.** Effect of genetic cargo on plaque morphology. *Escherichia coli* strain EV36 was mixed with appropriately diluted phage K1F wt (A), K1Fe6.7::NnLuc (B) and K1Fe14::NnLuc (C), and plated in soft agar on LB plates, followed by overnight incubation at 37°C.
**Figure S3.** Effect of genetic modification on plaque radius. Values are means of 5–10 plaques, error bars represent standard deviations. The asterisk marks a significant difference compared to K1F at a level of *p* <0.05, using a two‐tailed, unpaired *t*‐test.

## Data Availability

The data that support the findings of this study are openly available in Figshare at https://figshare.com/, reference number DOI: 10.6084/m9.figshare.27377133.

## References

[1] O'Neill J . Antimicrobial resistance: tackling a crisis for the health and wealth of nations. Review on Antimicrobial Resistance; 2014.

[2] Ballesté E et al. Bacteriophages in sewage: abundance, roles, and applications. FEMS Microbes. 2022;3:xtac009. doi:10.1093/femsmc/xtac009 37332509 PMC10117732

[3] Elahi Y , Nowroozi J , Fard RMN . Isolation and characterization of bacteriophages from wastewater sources on *Enterococcus* spp. isolated from clinical samples. Iran J Microbiol. 2021;13(5):671‐677. doi:10.18502/ijm.v13i5.7434 34900165 PMC8629828

[4] Runa V , Wenk J , Bengtsson S , Jones BV , Lanham AB . Bacteriophages in biological wastewater treatment systems: occurrence, characterization, and function. Front Microbiol. 2021;12:730071. doi:10.3389/fmicb.2021.730071 34803947 PMC8600467

[5] Singh A , Glass N , Tolba M , Brovko L , Griffiths M , Evoy S . Immobilization of bacteriophages on gold surfaces for the specific capture of pathogens. Biosens Bioelectron. 2009;24(12):3645‐3651. doi:10.1016/j.bios.2009.05.028 19520565

[6] Kretzer JW , Schmelcher M , Loessner MJ . Ultrasensitive and fast diagnostics of viable *Listeria* cells by CBD magnetic separation combined with A511::luxAB detection. Viruses. 2018;10(11):626. doi:10.3390/v10110626 30428537 PMC6266503

[7] Kunstmann S et al. Bacteriophage Sf6 Tailspike protein for detection of *Shigella flexneri* pathogens. Viruses. 2018;10(8):431. doi:10.3390/v10080431 30111705 PMC6116271

[8] Zhang H , Buttaro BA , Fouts DE , Sanjari S , Evans BS , Stevens RH . Bacteriophage φEf11 ORF28 endolysin, a multifunctional lytic enzyme with properties distinct from all other identified *Enterococcus faecalis* phage endolysins. Appl Environ Microbiol. 2019;85(13):e00555‐19. doi:10.1128/AEM.00555-19 30979842 PMC6581165

[9] Chibli H , Ghali H , Park S , Peter Y‐A , Nadeau JL . Immobilized phage proteins for specific detection of staphylococci. Analyst. 2014;139(1):179‐186. doi:10.1039/c3an01608k 24255915

[10] Choi Y‐J , Kim S , Kim J . Development of a magnetic bead‐based method for specific detection of *Enterococcus faecalis* using C‐terminal domain of ECP3 phage endolysin. J Microbiol Biotechnol. 2023;33(7):964‐972. doi:10.4014/jmb.2302.02033 37164751 PMC10394340

[11] Walcher G , Stessl B , Wagner M , Eichenseher F , Loessner MJ , Hein I . Evaluation of paramagnetic beads coated with recombinant *Listeria* phage endolysin‐derived cell‐wall‐binding domain proteins for separation of *Listeria* monocytogenes from raw milk in combination with culture‐based and real‐time polymerase chain reaction‐based quantification. Foodborne Pathog Dis. 2010;7(9):1019‐1024. doi:10.1089/fpd.2009.0475 20500083

[12] Foddai ACG , Grant IR . A novel one‐day phage‐based test for rapid detection and enumeration of viable *Mycobacterium avium* subsp. paratuberculosis in cows' milk. Appl Microbiol Biotechnol. 2020;104(21):9399‐9412. doi:10.1007/s00253-020-10909-0 32970181 PMC7567713

[13] Meile S et al. Engineered reporter phages for detection of *Escherichia coli*, *Enterococcus*, and *Lebsiella* in urine. Nat Commun. 2023;14(1):4336. doi:10.1038/s41467-023-39863-x 37474554 PMC10359277

[14] Molineux I . No syringes please, ejection of phage T7 DNA from the virion is enzyme driven. Mol Microbiol. 2001;40(1):1‐8. 11298271 10.1046/j.1365-2958.2001.02357.x

[15] Swanson NA et al. Expression and purification of phage T7 ejection proteins for cryo‐EM analysis. STAR Protoc. 2021;2(4):100960. doi:10.1016/j.xpro.2021.100960 34825220 PMC8605104

[16] Nelson TJ , Zhao J , Stains CI . Utilizing split‐NanoLuc luciferase fragments as luminescent probes for protein solubility in living cells. Methods Enzymol. 2019;622:55‐66. doi:10.1016/bs.mie.2019.02.003 31155065 PMC6567984

[17] Pósfai G et al. Emergent properties of reduced‐genome *Escherichia coli* . Science. 2006;312(5776):1044‐1046. doi:10.1126/science.1126439 16645050

[18] Vimr ER , Troy FA . Regulation of sialic acid metabolism in Escherichia coli: role of N‐acylneuraminate pyruvate‐lyase. J Bacteriol. 1985;164(2):854‐860. doi:10.1128/jb.164.2.854-860.1985 3902800 PMC214329

[19] Sambrook J . Molecular Cloning: a Laboratory Manual. 3rd ed. Cold Spring Harbor Laboratory Press; 2001.

[20] Blattner FR , Fiandt M , Hass KK , Twose PA , Szybalski W . Deletions and insertions in the immunity region of coliphage lambda: revised measurement of the promoter‐startpoint distance. Virology. 1974;62(2):458‐471. doi:10.1016/0042-6822(74)90407-3 4432374

[21] Quan J , Tian J . Circular polymerase extension cloning of complex gene libraries and pathways. PLoS One. 2009;4(7):e6441. doi:10.1371/journal.pone.0006441 19649325 PMC2713398

[22] Moller‐Olsen C , Ho S , Shukla R , Feher T , Sagona A . Engineered K1F bacteriophages kill intracellular *Escherichia coli* K1 in human epithelial cells. Sci Rep. 2018;8:17559. 30510202 10.1038/s41598-018-35859-6 PMC6277420

[23] Fehér T et al. Competition between transposable elements and mutator genes in bacteria. Mol Biol Evol. 2012;29(10):3153‐3159. doi:10.1093/molbev/mss122 22527906 PMC3457772

[24] Martel B , Moineau S . CRISPR‐Cas: an efficient tool for genome engineering of virulent bacteriophages. Nucleic Acids Res. 2014;42(14):9504‐9513. doi:10.1093/nar/gku628 25063295 PMC4132740

[25] Kiro R , Shitrit D , Qimron U . Efficient engineering of a bacteriophage genome using the type I‐E CRISPR‐Cas system. RNA Biol. 2014;11(1):42‐44. doi:10.4161/rna.27766 24457913 PMC3929423

[26] Fehér T , Karcagi I , Blattner FR , Pósfai G . Bacteriophage recombineering in the lytic state using the lambda red recombinases. J Microbial Biotechnol. 2012;5(4):466‐476. doi:10.1111/j.1751-7915.2011.00292.x PMC3815324 21910851

[27] Molineux IJ . The T7 group. In: Calendar R , ed. The Bacteriophages. Oxford University Press; 2006.

[28] Lukyanov KA et al. Natural animal coloration can be determined by a nonfluorescent green fluorescent protein homolog. J Biol Chem. 2000;275(34):25879‐25882. doi:10.1074/jbc.C000338200 10852900

[29] Matz MV , Fradkov AF , Labas YA , et al. Fluorescent proteins from nonbioluminescent Anthozoa species. Nat Biotechnol. 1999;17(10):969‐973. doi:10.1038/13657 10504696

[30] Mauri M , Vecchione S , Fritz G . Deconvolution of luminescence cross‐talk in high‐throughput gene expression profiling. ACS Synth Biol. 2019;8(6):1361‐1370. doi:10.1021/acssynbio.9b00032 31095908

[31] Hall M et al. Engineered luciferase reporter from a deep sea shrimp utilizing a novel imidazopyrazinone substrate. ACS Chem Biol. 2012;7(11):1848‐1857. 22894855 10.1021/cb3002478 PMC3501149

[32] Zhao J , Nelson T , Vu Q , Truong T , Stains C . Self‐assembling NanoLuc luciferase fragments as probes for protein aggregation in living cells. ACS Chem Biol. 2015;11(1):132‐138. 26492083 10.1021/acschembio.5b00758 PMC5110118

[33] Liyanagedera SBW et al. SpyPhage: a cell‐free TXTL platform for rapid engineering of targeted phage therapies. ACS Synth Biol. 2022;11(10):3330‐3342. doi:10.1021/acssynbio.2c00244 36194543

[34] Studier FW . Bacteriophage T7. Science. 1972;176(4033):367‐376. doi:10.1126/science.176.4033.367 4554613

[35] Bingen E et al. Bacterial counts in cerebrospinal fluid of children with meningitis. Eur J Clin Microbiol Infect Dis. 1990;9(4):278‐281. doi:10.1007/BF01968060 2112465

[36] Avramucz A , Wheatley J , Fehér T , Amaee R . Rapid, expression‐free bacteriophage‐based specific detection of target bacteria by conditional release of encapsidated reporter molecules. Research Square. 2025. doi:10.21203/rs.3.rs-5283843/v1 PMC13490980 42626106

