# Supplementary material for: Rapid, expression‐free bacteriophage‐based specific detection of target bacteria by conditional release of encapsidated reporter molecules
Source: Bioeng Transl Med. 2026 Aug 20:e70171. Online ahead of print. doi: 10.1002/btm2.70171 (PMC13490980; doi:10.1002/btm2.70171)
Supplement: Supplementary file 1 [file BTM2-9999-e70171-s001.docx]

Supplementary Material

# 1. Supplementary Methods

## 1.1. Donor plasmid construction

To generate the pSBC3g6.7::NnLuc donor plasmid, we started out with an older donor plasmid, pSBC3 [1], which carries a GFP gene flanked by homology arms that direct it to the 3’ end of g10b, the minor capsid protein of K1F. First, plasmid pSBC3 was PCR-amplified to exclude GFP and add TGA to 3’ end of g10b using primers pSBC3fw and pSBC3rev, using Q5 polymerase and 60 ^o^C annealing to obtain a 4320 bp product. Second, NnLuc was PCR amplified from NnLuc GeneStrands using primers NnLuc67Fw and NnLucRev using Q5 polymerase and 60 ^o^C annealing to obtain a 240 bp product. Third, gene g6.7 was amplified from 100-fold diluted K1F phage lysate using primers g6.7fw and g6.7rev using Q5 polymerase and 60 ^o^C annealing to obtain a 255 bp product. This PCR product includes the promoter of g6.7. The three PCR products were assembled in a Circular Polymerase Extension Cloning (CPEC) reaction [2] using Q5 polymerase and 60 ^o^C annealing. The product was purified using the Gel/PCR DNA Isolation System from Thermo scientific, and electroporated into *E. coli* MDS42, followed by plating on LB + Ampicillin agar plates. Colonies were screened by colony PCR using primers VF2 + NnLucCheckRev, using Taq polymerase and 57 ^o^C annealing to obtain a 609 bp product, and VR2 + NnLucCheckFw, using Taq polymerase and 57 ^o^C annealing to obtain a 350 bp product. Double positive colonies were grown for plasmid preparation. The resulting plasmids were sequence-verified by Sanger sequencing using VF2 and VR2. If both were correct, the plasmid was defined as pSBC3g6.7::NnLuc.

To construct pSBC3g14::NnLuc, the above procedure was repeated, with two changes: i) replacing the g6.7fw and g6.7rev primer pair with g14fw and g14rev, which give a 641 bp PCR product on the K1F genome; ii) replacing NnLuc67Fw with NnLuc14Fw. The NnLuc14Fw + NnLucRev PCR yields a 259 bp product. CPEC, purification, transformation and colony PCR was done identically, as above, but the correct PCR product of VF2 + NnLucCheckRev in this case is 976 bp long.

## 1.2. Cloning of C-terminal nanoluciferase (CnLuc) gene for bacterial expression

The DNA encoding the C-terminal part of nanoluciferase (CnLuc) protein was ordered from IDT as a GeneBlock. The CnLuc GeneBlock was PCR-amplified using primers CnLucFw and CnLucRev2 using Q5 polymerase, 60 ^o^C annealing. This PCR product was used as a template for a second round of PCR, using primers RBSfw and CnLucRev using Q5 polymerase, 60 ^o^C annealing. This product is the insert fragment (an RBS followed by the CnLuc gene).

The pET24dihyd_his_tetR plasmid was PCR amplified, excluding the ‘dihyd’ gene while keeping the hexahistidine tag and the stop codon using primers pET24CnLucFw and pET24CnLucRev by Q5 polymerase, 60 ^o^C annealing. This 5208 bp product is the vector fragment.

The insert and the vector fragments were fused using CPEC. The product was purified using the Gel/PCR DNA Isolation System from Thermo scientific, and electroporated into *E. coli* MDS42, followed by plating on LB + Chloramphenicol agar plates. Colonies were screened by colony PCR using primer pairs pET24CheckFW + CnLucRevCheck (585 bp) and pET24CheckRev + CnLucFWcheck (678 bp), both done using Taq polymerase at 57 ^o^C annealing temperature. The resulting plasmids were sequence-verified by Sanger sequencing using pET24CheckFW and pET24CheckRev. If both were correct, the plasmid was defined as pET24CnLuc_his.

In order to gain a plasmid to be used in the target bacterial strain, EV36, an additional plasmid was constructed by PCR using the pZA31CFPtetR plasmid [3] as a basis. The plasmid was PCR amplified, excluding the ‘CFP’ gene using primers pZALucFw2 and pZALucRev by Q5 polymerase, 60 °C annealing. This 2787 bp product is the vector fragment.

The insert fragment was amplified from the previously constructed, sequence-verified pET24CnLuc_his transferring its RBS, hexahistidine tag, CnLuc gene and stop codon. PCR amplification was carried out using primers RBSFw and CnLucRev3 by Q5 polymerase, 60 ^o^C annealing.

The insert and the vector fragments were fused using CPEC. The product was purified using the Gel/PCR DNA Isolation System from Thermo scientific, and electroporated into *E. coli* MDS42, followed by plating on LB + Chloramphenicol agar plates. Colonies were screened by colony PCR using primer pairs pZAcheckFW + CnLucRevCheck (659 bp) and pZACheckRev + CnLucFWcheck (663 bp), both done using Taq polymerase at 57 ^o^C. The resulting plasmids were sequence-verified by Sanger sequencing using pZACheckFW and pZA31CheckRev. If both were correct, the plasmid was defined as pZA31CnLuc_his_tetR.

## 1.3. Phage titering and plaque analysis

Phage titres were determined as described earlier [4]. Briefly, 10-fold dilution series were made of each phage using buffer Φ80+ in a 96-well microtiter plate, and 10 μl from each well was plated on a lawn of *E. coli* EV36. The dilution that yielded individual plaques were re-plated, this time by mixing 100 μl of the phage with 3 ml of molten soft agar (kept at 42 ^o^C), containing 10^7^ *E. coli* EV36 cells. The mixture was poured onto LB plates, followed by overnight incubation at 37 ^o^C prior to plaque counting and morphological analysis. Plaque diameters were measured using the OpenCFU software [5].

## 1.4. Purification of recombinant phages

- Pellet freshly made phage lysates in a microcentrifuge for 2 min at 10,000 g. Recover the supernatant
- Pass the supernatant through a 0.22 μm syringe filter (Filtropur S 0.2, Sarstedt, Nümbrecht, Germany)
- Load the filtrate onto Amicon Ultra regenerated cellulose centrifugal filters (100 kDa cutoff, Merck-Millipore, Burlington, Massachusetts, USA)
- Centrifuge at room temperature at 4500 g until between 80 and 90% of the sample liquid had passed through the filters (usually 8-10 minutes using new filters)
- Dilute upper, concentrated sample back to original volume. Discard the ultrafiltrate
- Repeat the process for higher purity, if required

# 2. Supplementary Results

## Effect of genetic modification on phage phenotype

|  | titer (x109) |
| --- | --- |
| K1F wt | 9.66667 |
| K1Fe6.7::NnLuc | 7.66667 |
| K1Fe14::NnLuc | 10.3333 |

**Figure S1**. Comparison of phage titres obtained with various phages. The three phages were grown on *E. coli* EV36 until complete lysis was observed. Values are means of three dilution replicates, error bars represent standard deviations.

**Figure S2**. Effect of genetic cargo on plaque morphology. *E. coli* strain EV36 was mixed with appropriately diluted phage K1F wt (A), K1Fe6.7::NnLuc (B) and K1Fe14::NnLuc (C), and plated in soft agar on LB plates, followed by overnight incubation at 37 ^o^C.

|  | plaque radius (AU) |
| --- | --- |
| K1F | 55.4 |
| K1Fe6.7::NnLuc | 47.6 |
| K1Fe14::NnLuc | 44.1667 |

**Figure S3**. Effect of genetic modification on plaque radius. Values are means of 5-10 plaques, error bars represent standard deviations. The asterisk marks a significant difference compared to K1F at a level of p<.05, using a two-tailed, unpaired t test.

**Bibliography**

1. Moller-Olsen C, Ho S, Shukla R, Feher T, Sagona A. Engineered K1F bacteriophages kill intracellular Escherichia coli K1 in human epithelial cells. Sci Rep. 2018;8:17559.

2. Quan J, Tian J. Circular polymerase extension cloning of complex gene libraries and pathways. PloS One. United States; 2009;4:e6441. https://doi.org/10.1371/journal.pone.0006441

3. Fehér T, Bogos B, Méhi O, Fekete G, Csörgo B, Kovács K, et al. Competition between transposable elements and mutator genes in bacteria. Mol Biol Evol. United States; 2012;29:3153–9. https://doi.org/10.1093/molbev/mss122

4. Fehér T, Karcagi I, Blattner FR, Pósfai G. Bacteriophage recombineering in the lytic state using the lambda red recombinases. Microb Biotechnol. United States; 2012;5:466–76. https://doi.org/10.1111/j.1751-7915.2011.00292.x

5. Geissmann Q. OpenCFU, a new free and open-source software to count cell colonies and other circular objects. PloS One. United States; 2013;8:e54072. https://doi.org/10.1371/journal.pone.0054072
